# Uncovering spatial and ecological variability in gap size frequency distributions in the Canadian boreal forest

**DOI:** 10.1038/s41598-020-62878-z

**Published:** 2020-04-08

**Authors:** Tristan R. H. Goodbody, Piotr Tompalski, Nicholas C. Coops, Joanne C. White, Michael A. Wulder, Marco Sanelli

**Affiliations:** 1 Faculty of Forestry, 2424 Main Mall, University of British Columbia, Vancouver, BC V6T 1Z4 Canada; 2 Canadian Forest Service (Pacific Forestry Center), Natural Resources Canada, 506 West Burnside Road, Victoria, BC V8Z 1M5 Canada

**Keywords:** Forest ecology, Forestry

## Abstract

Analyses characterizing canopy gaps are required to improve our understanding of spatial and structural variations in forest canopies and provide insight into ecosystem-level successional processes. Gap size frequency distributions (GSFD) are indicative of ecological processes and disturbance patterns. To date, GSFD in boreal forest ecosystems have not been systematically quantified over large areas using a single consistent data source. Herein we characterized GSFDs across the entirety of the Canadian boreal forest using transects of airborne laser scanning (ALS) data. ALS transects were representatively sampled within eight distinct Canadian boreal ecozones. Gaps were detected and delineated from the ALS-derived canopy height model as contiguous canopy openings ≥8 m^2^ with canopy heights ≤3 m. Gaps were then stratified by ecozone and forest type (i.e. coniferous, broadleaf, mixedwood, wetland-treed), and combinations thereof, and GSFDs were calculated for each stratum. GSFDs were characterized by the scaling parameter of the power-law probability distribution, lambda (λ) and Kolmogorov-Smirnov tests confirmed that GSFDs for each stratum followed a power-law distribution. Pairwise comparisons between ecozones, forest types, and combinations thereof indicated significant differences between estimates of λ. Scaling parameters were found to be more variable by ecozone (1.96–2.31) than by forest type (2.15–2.21). These results contrast those of similar studies done in tropical forest environments, whereby λ was found to be relatively consistent across a range of site types, geological substrates, and forest types. The geographic range considered herein is much larger than that of previous studies, and broad-scale patterns in climate, landforms, and soils that are reflected in the definition of unique ecozones, likely also influence gap characteristics.

## Introduction

Data characterizing the state and variability of large- and fine-scale forest disturbances is critical for evaluating forest dynamics and influencing current and future management strategies^1^. The need for these data is especially pertinent in extensively forested nations such as Canada, where boreal forests cover more than 270 Mha^2,3^, are predominantly remote, and are subject to seasonably variable and harsh climates^4^. Limited access to much of the Canadian boreal has constrained management intervention and industrial development^5^, leading to reliance on remotely sensed information to monitor, study, and generate synoptic data products for these ecosystems^6^. Generated using satellite based time-series, high-quality spatially explicit data detailing land cover^7^, disturbance^8,9^, and recovery^1,10^ have provided valuable insight into boreal forest dynamics and trends. These remote sensing based analyses serve to inform the ecological importance of biotic and abiotic disturbances, highlighting the implications of forest structural and physiological change at local to regional scales^11,12^.

Within the context of regional satellite based remote sensing studies, an abundance of research has focused on the prevalence and influence of spatially extensive abiotic and biotic disturbances such as stand replacing wildfires and non-stand replacing insect outbreaks^6,7,10,13–16^. Over boreal ecosystems, wildfire is recognized as a dominant driver for the spatial and structural conditions present, and have been shown to influence carbon and water dynamics^17–19^, create shifts in competitive wildlife use^20,21^, and in the long-term, rejuvenate ecosystem function through successional change^22–24^. Given the size of the Canadian boreal, substantial variability in fire regimes exist^8^, leading to differences in the manifestation and functional effect of fire events^25^.

In the absence of fire, other non-stand replacing disturbances are prevalent. Biotic disturbances including insect and fungal outbreaks, as well as abiotic events such as storm related wind throw and drought stress, result in widespread and important successional changes to forested ecosystems^11,26,27^. The role of these non-fire disturbances has been largely underemphasized, particularly in regards to their creation of canopy gaps and gap characteristics^28^. Canopy gaps are influential drivers of forest development^26,28^ and have been found to be highly variable in size^29^. Herein we refer to gaps as openings in the forest canopy originating from natural causes such as tree mortality, wind throw events, and branch fall^30^. Observed synchrony in the timing of gap formation suggests that landscape level events control gap dynamics^31,32^, with recent research indicating that their functional presence may not manifest prior to a few decades of development in boreal stands^33^.

Studies analyzing canopy gaps have largely been defined by a trade-off between the cost of data acquisition and the level of spatial and attributional detail for their delineation^34^. Until recently, canopy gaps were characterized via costly, time consuming, manual methodologies^35–37^, generally limiting extent and representation of research activity. The development of airborne laser scanning (ALS) and other remote sensing approaches has notably reduced barriers to characterizing canopy gaps over a range of forest environments^33,34,36,38–44^. The development of ALS has facilitated delineation of canopy gaps and their prevalence across forested landscapes^41^. Delineation of gaps is a straight-forward process, where minimum canopy height thresholds are established and used to delineate openings in ALS generated canopy height models (CHM)^34^. Following their delineation, gap attributes such as area, shape, and density are often derived. Vepakomma *et al*.^45,46^ for example investigated the potential to use multi-temporal ALS datasets in the eastern Canadian boreal forest to examine changes in gap size and shape over time. White *et al*.^34^ likewise tested the capacity of ALS to delineate canopy gaps in a coastal temperate rainforest in western Canada, outlining its enhanced accuracy in comparison to co-occurring digital aerial photogrammetric datasets.

A method for summarizing landscape level characteristics of canopy gaps using ALS delineated gaps is the calculation of gap size frequency distributions (GSFD), which describe the relative frequency of gaps across a range of gap size classes^35^. GSFDs are often found to follow a power-law distribution, wherein the negative slope resulting from plotting gap frequency against gap size classes on a log-log scale is characterized by lambda (λ), the scaling exponent of the power-law distribution^38,47^. In these studies λ has been used to compare GSFDs for areas with different geological substrates and forest types^41,48^. Values of λ have been theorized to have a range from 1.0–3.0 in tropical forests^38,42,48–50^, with a threshold of 2.0 being indicative of whether a forest is dominated by larger canopy gaps resulting from spatially extensive, stand-initiating disturbances (λ < 2.0), or smaller gaps, which may be indicative of low mortality, high growth dynamics (λ > 2.0) and small disturbances^38^.

To address the paucity of research focusing on boreal canopy gap characteristics and facilitate inter-comparisons of GSFDs from global forest types, we detected and delineated canopy gaps, and quantified and compared GSFDs within eight distinct ecological units (ecozones) and four forest types (coniferous, deciduous, mixedwood, wetland treed) in the boreal forest of Canada. To offer extensive spatial representation as well as required local forest structural detail, a 25,000 km network of ALS transect data were used, enabling a spatially explicit and consistent means of delineating canopy gaps, and consequently GSFDs across Canadian boreal regions.

The aims of this study were to investigate whether boreal GSFDs followed power-law distributions, and whether the relationship between gap size and frequency differed amongst ecozones and forest types. In order to address these aims we posed the following questions:

### Do gaps in boreal ecozones and forest types follow a power-law distribution?

Gap size frequency analyses within various tropical forest environments have found that power-law distributions are common^41,42,48^. As no similar broad-scale studies have been conducted within boreal forest environments, we had no *a priori* expectations as to the form of gap size frequency distributions.

### Do scaling exponents for power-law distributions (λ) differ amongst boreal ecozones and forest types?

The λ scaling exponent describes the relationship between gap size and frequency for power-law distributions. Previous studies in tropical forests have found minimal variation in scaling exponents across different sites, geological substrates, and forest types^41,48^. We therefore expected that λ values would not differ significantly among ecozones or forest types, confirming the consistency of scaling exponents across variable forest conditions.

In addition to these overarching research questions and hypotheses, we present descriptive statistics on gap size, shape, and density by ecozones and forest types. Finally, we considered GSFD for boreal forests with reference to values reported in the literature representing other forested regions with an aim to better understand similarities and differences in GSFDs and aid in the interpretation of our results in a broader context.

## Materials and methods

### Study area

The Canadian boreal forest is characterized by short cool summers, long cold winters, and is subject to variable temperature ranges^3^. Variations in climate as well as biotic and abiotic factors have led to the delineation of 6 boreal terrestrial ecozones^51^, the largest of which, the Taiga and Boreal Shield, are often divided into eastern and western sub-units to better reflect their eco-climatic conditions^1,10^. Proportions of managed land within ecozones vary, with Taiga Plains, Taiga Shield East and West, and Hudson Plains having <20%, Boreal Shield East and West <70%, and Boreal Plains <90% area considered to be managed^1^.

### Airborne laser scanning data

**Figure 1:**
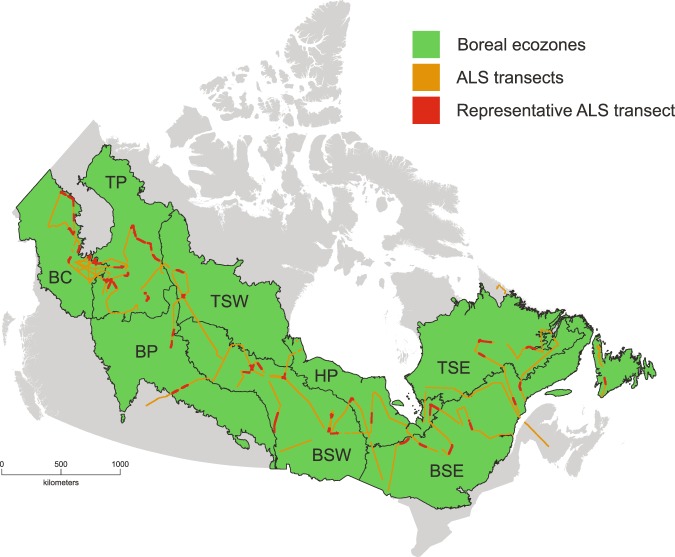
Study area map of Canadian boreal ecozones (Boreal Cordillera (BC), Taiga Plain (TP), Boreal Plain (BP), Taiga Shield West/East (TSW/E), Hudson Plain, and Boreal Shield West/East (BSW/E) (green), ALS transects acquired in 2010 (orange), and representative sections of transect used for canopy gap delineation (red).

Small-footprint, discrete return ALS data is an exhaustively researched technology with proven potential for characterizing forest structure and improving forest inventory information at multiple scales^52,53^. To leverage the benefits of this technology for canopy gap delineation, ALS data were acquired in the summer of 2010 by the Canadian Forest Service in collaboration with the Canadian Consortium for LiDAR Environmental Applications Research and the Applied Geomatics Research Group. Thirty-four transects averaging 700 km in length (~25,000 km in total) were acquired over all Canadian boreal forest ecozones (Fig. 1). Acquisition parameters for ALS transects are detailed in Table 1.

**Table 1:** ALS acquisition parameters.

Parameter	Description
Sensor	Optech ALTM 3100
Number of Transects	34
Average Transect Length	700 km
Average Aircraft Speed	277 km/h
Data Acquisition Height	450–1900 m AGL
Minimum Swath Width	>400 m
Pulse Repetition Rate	70 kHz
Beam Divergence	0.3 mrad
Fixed Scan Angle	15°
Point Density	2.8 pt./m^2^

ALS transects were processed using the LAStools^54^ programming suite, which enables fast and efficient reading, writing, and manipulation of ALS point clouds. In order to compute CHMs and canopy cover (% of ALS points > 2 m) layers for all ALS transects, point clouds were first divided into 1 km^2^ tiles using *lastile* and filtered to remove noise using *lasnoise*. Points were then classified as ground or non-ground points using *lasground* to extract a digital terrain model, and height normalized using *lasheight*. Normalized point clouds were used to generate CHMs at a 2 m resolution and canopy cover at 20 m resolution using *lascanopy*.

### Selection of representative ALS transect sections

To ensure that areas of analysis encompassed as much variability in vegetation as possible, the MODIS MOD17A3 (https://lpdaac.usgs.gov/products/mod17a3v055/) gross primary productivity (GPP) product was used to delineate samples of ALS transects that were most representative of corresponding ecozones. MODIS derived GPP are well established to provide reliable vegetation productivity estimates^55^. Given that fluctuations in temperature and vegetation condition influence productivity estimates, individual years may not be representative of long-term productivity. To account for these variations, annual GPP estimates from 2005–2010 were averaged to align productivity with estimates of vegetation structure. Delineation of representative ALS transect sections was conducted using a probability distribution function where 30% of averaged MODIS GPP pixels intersecting ALS transects were randomly sampled with replacement over 10,000 iterations. Kolmogorov-Smirnov tests were then used to assess whether significant (*a* = *0.05*) differences between bootstrapped GPP sample distributions and distributions of GPP for each ecozone existed. Transect sections maximizing the fit between ecozone GPP distributions and the sample distribution were considered representative of the ecozone and were selected for further processing. Representative transect sections used in this study are open data and include height percentiles, canopy cover, and coefficient of variation of height^56^.

### Canopy gap delineation

To isolate forested areas within representative ALS transect sections, only areas meeting the definition for forest used by the Food and Agriculture Organization (FAO) of the United Nations were used in our analysis^57^ (i.e. areas ≥ 0.5 ha, canopy cover ≥ 10%, and height ≥ 5 m). CHMs and canopy cover (% of ALS pts > 2 m) layers were used to delineate areas within transects meeting threshold specifications.

**Figure 2:**
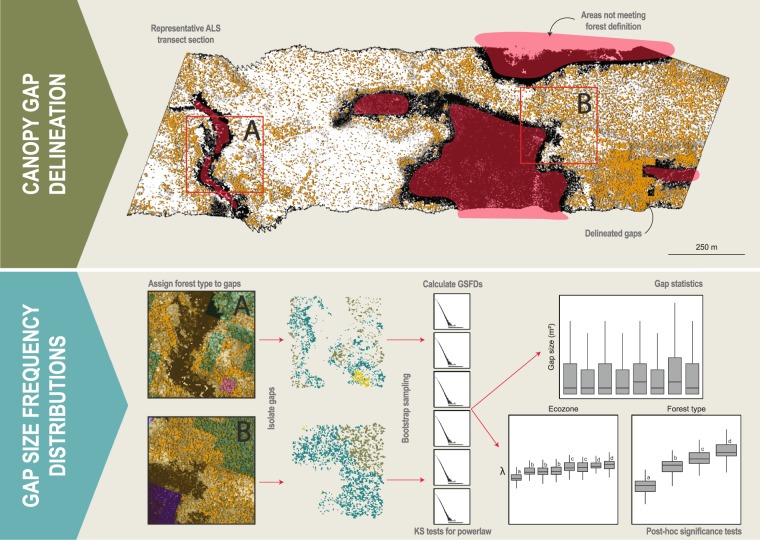
Graphical description of applied canopy gap delineation and gap size frequency distribution (GSFD) methodology.

Following delineation of forested areas within representative ALS transects, a height threshold of 3 m^40^ was applied to CHMs to differentiate between gap and no-gap pixels. All pixels with heights ≤ 3 m were identified as gaps (Fig. 2). Connected gap pixels were considered single contiguous gaps. A maximum canopy gap size was not defined as has been presented in previous literature^34^ in order to include all gaps within delineated forested areas regardless of their area coverage and complexity of shape. Following delineation, each gap was attributed a respective ecozone, as well as assigned a forest type (coniferous, broadleaf, mixedwood, or wetland-treed) based on the disturbance informed Virtual Land Cover Engine (VLCE), a Landsat derived, spatially extensive, temporally dense, and flexible framework for mapping land cover^7^.

In order to characterize properties of detected gaps, a number of spatial characteristics including gap area, shape index, and distance to nearest gap were computed. Shape index to characterize gap shape complexity was calculated using Eq. 1.

1 $${\rm{shape}}\,{\rm{index}}=\frac{{\rm{perimeter}}}{2\ast {({\rm{\pi }}\ast {\rm{area}})}^{0.5}}$$

A shape index of 1 describes a perfectly circular gap. As shape index increases, the gap boundary becomes increasingly complex. Distance to nearest gap indicates gap density and was calculated as the distance between the bounding boxes of a gap and its closest neighbour.

### Gap size frequency distributions

Gap size frequency distributions for all boreal ecozones and forest types were calculated using the approach of Hanel *et al*.^58^, which employs Bayesian parameter estimation utilizing maximum likelihood estimators that calculate λ across the entire range of reasonably accessible values^59,60^. This method also integrates a two-sided Kolmogorov-Smirnov goodness of fit test to determine whether GSFDs follow a power-law distribution. A key parameter when calculating GSFDs is the minimum included gap size. In this analysis we tested the impact of minimum gap size by including gaps ranging from 4 m^2^ (i.e. a single 2 m CHM cell) to a maximum of 80 m^2^. The number of samples included in each bootstrap (500, 1000, 2000, 5000, and 10000) was also assessed.

A smallest gap size of 8 m^2^ with 5000 samples per bootstrap iteration was chosen due to consistency between bootstrapped λ estimates. These parameters were used for quantifying GSFDs for ecozones, forest type, and their combinations. Estimates of λ were not generated where the minimum number of gap samples was not met.

Bootstrapped estimates of λ provided distributions that facilitated the two-sided Kolmogrov-Smirnov test for each ecozone, forest type, and ecozone/forest type combination to determine whether distributions conform to that of a power-law. Kruskal-Wallis and Dunn’s post-hoc tests were applied to assess whether ecozones, forest type, and their pairings showed significantly (*a* = *0.05*) different λ estimates.

## Results

### Delineated gap features

**Figure 3:**
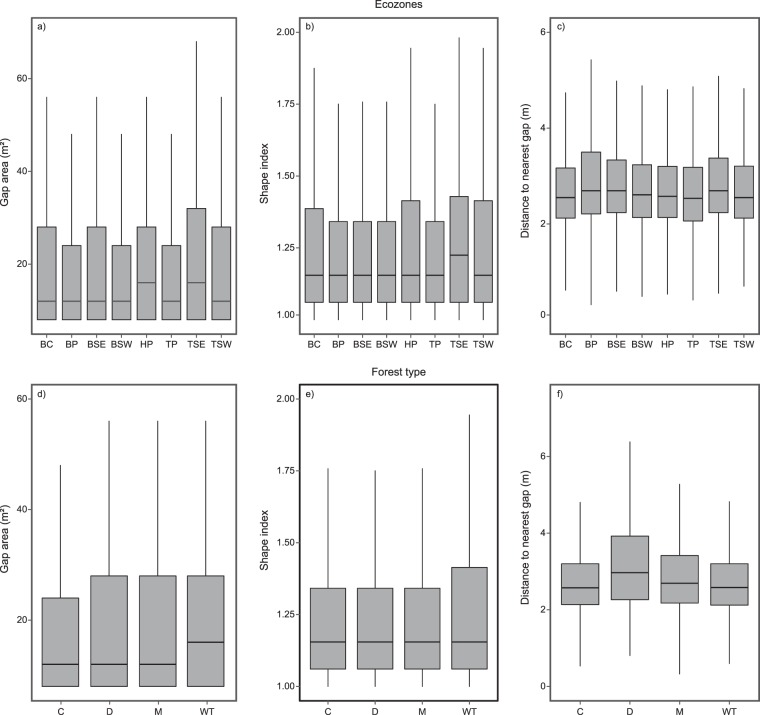
Summaries of gap area (**a,d**) (m^2^), shape index (**b,e**), and distance to nearest gap (**c,f**) (m) for ecozones (Boreal Cordillera (BC), Boreal Plains (BP), Boreal Shield East (BSE), Boreal Shield West (BSW), Hudson Plain (HP), Taiga Plain (TP), Taiga Shield East (TSE), and Taiga Shield West (TSW)) and forest types (Coniferous (C), Deciduous (D), Mixedwood (M), and Wetland-treed (WT)).

A total of 1,037,358 gaps were included with a mean gap area of 57.7 m^2^. Hudson Plain and Taiga Shield East ecozones had the largest median gap sizes relative to other ecozones (Fig. 3a). Taiga Shield East had the largest inter-quartile range for gap area and shape index (Fig. 3b). Distance to nearest gap was greatest for deciduous stands, however was relatively consistent across forest types with a coefficient of variation of 38.3% (Fig. 3c). Wetland-treed gaps were found to have a higher median gap area and larger interquartile range for shape index (Fig. 3d,e). Coniferous gaps were found to have the smallest inter-quartile range for gap size. Deciduous gaps had the largest median, and greatest variability, in distance to nearest gap (Fig. 3f).

### Gap size frequency distributions

**Figure 4:**
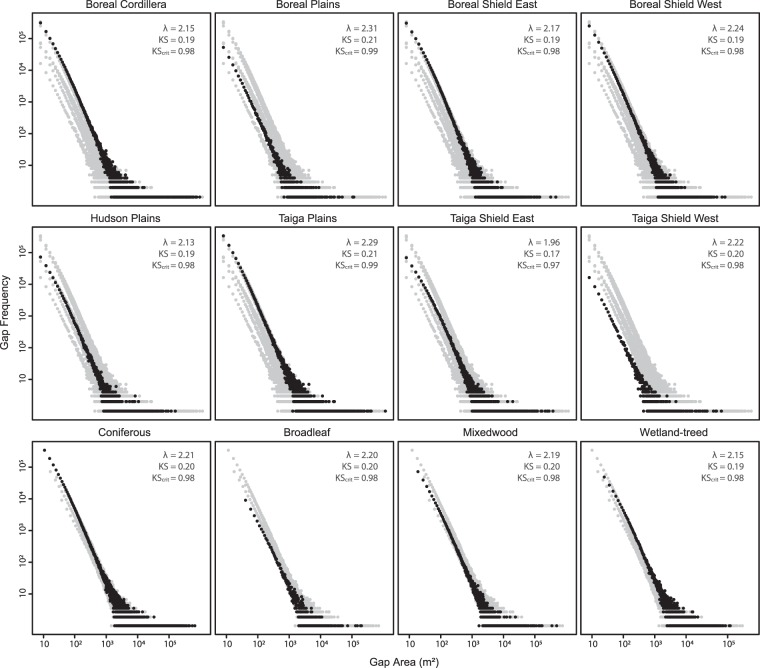
Gap size frequency distributions (GSFD) in eight boreal ecozones and four forest types. Dark grey markers represent the GSFD for each ecozone/forest type, while light grey markers represent GSFD for all ecozones/forest types combined. Axes are logarithmic. Kolmogorov-Smirnov test statistics (KS), critical test statistics (KScrit), and λ are the mean values from iterative bootstrapping.

Two-sided Kolmogorov-Smirnov tests indicated that the GSFDs for all ecozones, forest types and combinations thereof conformed to a power-law distribution (Fig. 4). Mean λ for all ecozones was 2.18, with the Boreal Plains having the highest (λ = 2.31) and Taiga Shield East having the lowest (λ = 1.96). All ecozone λ estimates were greater than 2.0, except Taiga Shield East. Mean λ for forest types were highest for conifers (λ = 2.21) and lowest for wetland-treed (λ = 2.15; Table 2). All ecozones paired with coniferous forest types had sufficient gap samples to conduct bootstrapping. Taiga Plains, Boreal Plains, and Boreal Shield East ecozones were the only ecozones with sufficient gap samples to calculate λ for all ecozone-forest type combinations (Table 2).

**Table 2:** Gap size frequency distribution (GSFD) parameters calculated for gaps in eight ecozones stratified into four forest types: coniferous, deciduous, mixedwood, and wetland-treed.

Ecozone	Coniferous			Broadleaf			Mixedwood			Wetland-treed		
	λ	KS	KScrit	λ	KS	KScrit	λ	KS	KScrit	λ	KS	KScrit
*Forest type mean*	2.21	0.20	0.98	2.20	0.20	0.98	2.19	0.20	0.98	2.15	0.19	0.98
Boreal Cordillera	2.15	0.19	0.98	—	—	—	—	—	—	—	—	—
Boreal Plains	2.37	0.22	0.99	2.28	0.21	0.99	2.22	0.21	0.99	2.23	0.2	0.98
Boreal Shield East	2.18	0.19	0.98	2.18	0.19	0.98	2.17	0.19	0.98	2.09	0.18	0.98
Boreal Shield West	2.3	0.21	0.98	—	—	—	2.17	0.19	0.98	2.15	0.19	0.98
Hudson Plains	2.15	0.19	0.98	—	—	—	—	—	—	2.12	0.19	0.98
Taiga Plains	2.3	0.2	0.99	2.11	0.2	0.98	2.31	0.21	0.99	2.34	0.21	0.99
Taiga Shield East	1.95	0.17	0.97	—	—	—	1.96	0.17	0.97	2.05	0.18	0.98
Taiga Shield West	2.22	0.2	0.98	—	—	—	—	—	—	—	—	—

Kolmogorov-Smirnov test statistics (KS), and critical test statistics (KScrit) were generated to determine whether distributions followed power-law.

**Figure 5:**
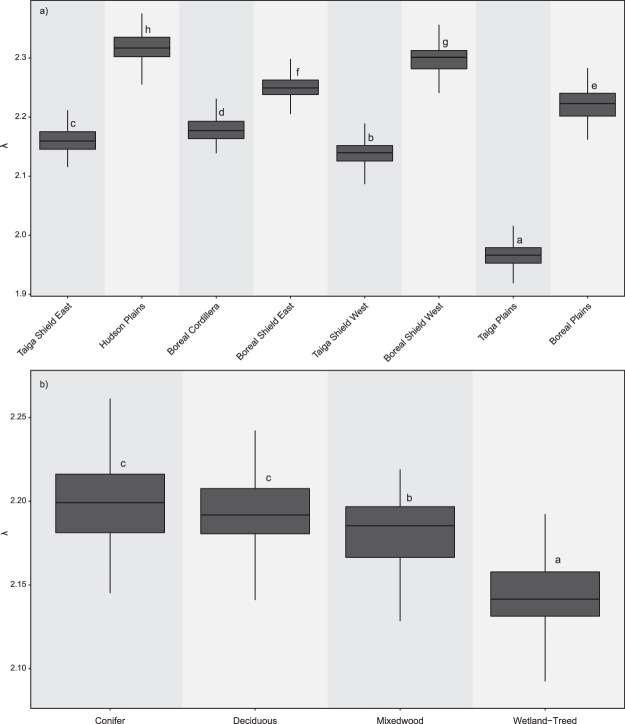
Comparison of bootstrapped distributions of λ for (**a**) ecozone (Boreal Cordillera (BC), Boreal Plains (BP), Boreal Shield East (BSE), Boreal Shield West (BSW), Hudson Plain (HP), Taiga Plain (TP), Taiga Shield East (TSE), and Taiga Shield West (TSW)), and (**b**) forest type (Coniferous (C), Deciduous (D), Mixedwood (M), and Wetland-treed (WT). Letters above boxplots indicate Dunn’s post-hoc test results denoting whether distributions were significantly different (a = 0.05).

Kruskall-Wallis and Dunn’s post-hoc tests were conducted for ecozones, forest types, and combinations thereof. All ecozones were found to have significantly different λ distributions (Fig. 5a). Forest types except conifer and deciduous had significantly different distributions of λ (Fig. 5b).

**Figure 6:**
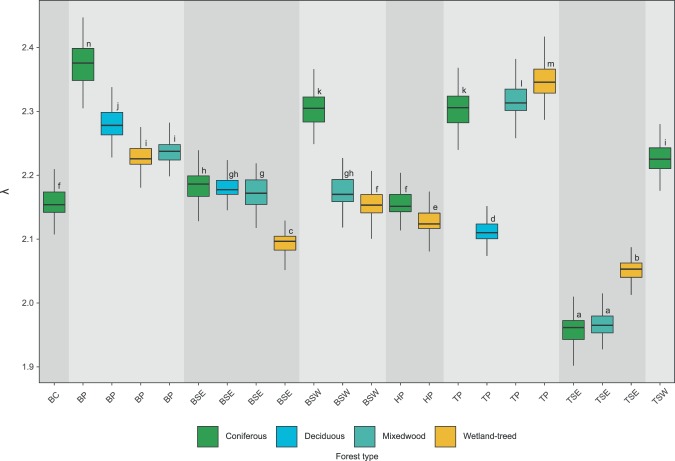
Comparison of bootstrapped distributions of λ for ecozone-forest type pairings. Letters above each boxplot indicate Dunn’s post-hoc test results denoting whether distributions were significantly different (a = 0.05) from one another. Boreal Cordillera (BC), Boreal Plains (BP), Boreal Shield East (BSE), Boreal Shield West (BSW), Hudson Plain (HP), Taiga Plain (TP), Taiga Shield East (TSE), and Taiga Shield West (TSW).

Combinations of ecozone and forest type showed a range of λ results (Fig. 6). Gaps within coniferous and mixedwood forests in the Taiga Shield East ecozone were found to have the two lowest overall λ values. These two pairings were the only distributions with λ values below 2.0. Distributions for coniferous Hudson Plains, wetland-treed Boreal Shield West, and coniferous Boreal Cordillera shared similar distributions. Coniferous Taiga Shield West and both deciduous and mixedwood Boreal Plains shared similar distributions of λ. The coniferous Boreal Plains pairing was found to have the highest estimated λ distribution.

## Discussion

In this study, we delineated canopy gaps within representative sections of ALS transects to compute GSFDs across a range of forest conditions in Canadian boreal forest ecozones. We found that the number and size of gaps, as well as GSFDs were different for ecozones, forest types, and their pairing. Our results represent the first spatially extensive quantification of GSFDs within a boreal forest context, complementing that of existing studies primarily focused on tropical forest ecosystems. The results presented herein can provide insight for investigations of boreal forest dynamics and improve understanding of similarities and differences amongst global forest systems. Comparisons between boreal and tropical GSFDs, with reference to ecological foundations and gap formation theory, can inform our understanding of forest dynamics in these different regions.

The Canadian boreal, though variable by region, is dominated by stand replacing wildfires^1^ with highly variable patch sizes^61^. Fires create landscape-level heterogeneity of forest structure, which are subject to smaller scale insect attacks or individual/group mortality events in their absence. Tropical forests such as those found in the Amazon region have markedly different climatic conditions, species composition, structure, and disturbance regimes from boreal forests. Espírito-Santo *et al*.^62^ estimated that small disturbances (<0.1 ha) are much more probable than large events (≥5 ha) in the Amazon region. Low probabilities of large disturbances in these forests contrast markedly to Canadian boreal ecozones where historically, wildfire has burned an average of 1.5 Mha annually (based on data representing 1985–2010)^1^. These spatially extensive disturbances were excluded from this analysis. Patches impacted by wildfire in Canada’s boreal are commonly 2–5 km^2^ in size (with some exceeding 1000 km^2^)^8^. Any recent wildfires (i.e. since 1985) within the bounds of ALS transects that did not meet the required thresholds for forest height, canopy cover, and minimum size were not considered in our analysis. With this context in mind, we discuss results and outcomes of this study with reference to our initial research questions and hypotheses.

### Do gaps in boreal ecozones and forest types follow a power-law distribution?

Similar to results reported across a range of conditions in tropical forests, we found that gap size frequency in boreal forests followed a power-law distribution. Identifying power-law distributions for ecological features such as canopy gaps provides insight into the nature of gap formation processes such as tree mortality and local stand level disturbance. Changes in gap size were found to result in a similarly proportioned change in gap frequency, and that small canopy gaps were more prevalent than large canopy gaps within boreal forest ecozones and forest types.

### Do scaling exponents for power-law distributions (λ) differ amongst boreal ecozones and forest types?

Our results confirmed that λ varied more amongst ecozones than forest types. Ecozones were all found to exhibit significant differences in λ, and had greater variability in scaling exponents. Forest types were found to exhibit less variability in λ, and significant differences in λ were not found between coniferous and deciduous forest types.

A commonly implemented heuristic in tropical forests is that GSFDs with λ values below 2.0 are dominated by larger gaps, created through spatially extensive disturbances, whereas GSFDs with λ values above 2.0 are dominated by smaller gaps and localized disturbance processes^38,41,47^. Tropical studies have reported λ values ranging from 1.1–1.6^38^ and 1.9–3.1^47^ in Amazonian forests, and 1.66–1.99^63^ and 1.8–2.6^48^ in Costa Rican and Hawaiian forests respectively. Asner *et al*.^41^, likewise computed GSFDs in the Peruvian Amazon for a range of canopy depth classes (≥1 and ≥20 m), geologic, topographic, and physiognomic descriptors with largely consistent findings of λ values between 1.70–2.03. Within the context of these previous studies in tropical forest environments, results for λ presented herein for the Canadian boreal are 1.96–2.31 for ecozones and 2.15–2.21 for forest types. Scaling parameters were found to be relatively consistent across varying conditions in tropical forests^41,48^. However, we found significant differences in λ values among ecozones and some forest types, and greater variation among ecozones than forest types. Herein, the geographical range across which we evaluated gap size frequency distributions was much broader than that of past studies, suggesting that variations in climate, landforms, and soil types among others, which are inherent to the definition of Canada’s terrestrial ecozones, also influence gap characteristics.

Gap formation theory helps to support similarities among forest type GSFDs. In the event of tree mortality and windthrow, larger, higher biomass trees are more likely to result in larger canopy gaps^64,65^, and corresponding lower λ values. Boreal forests are predominantly short, have low diversity, and are comprised of regular crown shapes^66^. Short growing seasons in boreal forests reduce annual growth potential, generally resulting in smaller trees^67^. Reasonably similar estimates of scaling parameters among boreal forest types, especially conifer, deciduous, and mixedwood stands indicate similarities in the distribution of canopy gap sizes. Of all forest types considered, wetland-treed forests had the lowest values of λ and therefore greatest prevalence of larger canopy gaps. Larger proportions of these gaps are likely driven by prevalence of surface water, high soil moisture, and low nutrient edaphic conditions common to wet-land forest environments. Values of λ below 2.0 were also found for conifer and mixed-wood forests in the Taiga Shield East ecozone. This region is known for its harsh long winters, abundance of water features, and shallow wet soils, potentially leading to more spatially scattered, lower density, forest stands. Larger median gap sizes and shape indices for both the Taiga Shield East ecozone and wetland-treed forest type further reinforce these findings.

In the tropics, relative consistency in GSFDs has been attributed to light availability. Studies have modeled how forest canopy gaps develop in tropical forests using cellular automata theory^68,69^, which addresses how complex systems approaching a critical state will self-organize into similar spatial patterns. To this end, tropical forest species have been found to exhibit opportunistic and plastic growth patterns forming intricate and irregular tessellations^70^. These growth patterns result in tree canopies filling most of the tri-dimensional canopy space, which explains the gap-scaling processes across landscapes within a particular region^41,70^. While there is still debate surrounding the high degree of scaling consistency in forest ecosystems, resource limitation such as light has been thought to be the cause of this homogeneity in tropical forests^41^.

Tropical and temperate forests experience drastic differences in light levels between gap and non-gap areas. In boreal forests, gap and non-gap areas experience similar light distributions likely due to the presence of small gaps that are fairly evenly spaced^71^. Power-law distributions for boreal GSFDs and distance to nearest gap metrics presented herein help to confirm this. Research in boreal and temperate forests found spatial patterns to be clustered in young stands and more regular in older aged stands^72,73^. In these forests, competition between tree species drives forest patterns away from spatial clustering towards regularity^74,75^. Trees are opportunistic in their growth and will fill available canopy space^76^. This crown plasticity is particularly pronounced in mixed-species forests^77^. In boreal forests, we posit that this opportunistic growth is the primary reason for consistency in scaling parameters among forest types.

## Conclusions

Findings from this study provide a useful baseline that quantifies the variability in GSFDs in the Canadian boreal forest. The relationship between gap size and frequency for all ecozones and forest types were found to fit a power-law distribution. Scaling parameters varied more by ecozone than by forest type, suggesting that broad-scale patterns in climate, landforms, and soils that are inherent to the definition of ecozones, also influence gap characteristics. Findings herein facilitate a comprehensive understanding and inter-comparison among global forest types that can help form the basis of additional hypotheses with respect to canopy gap size and frequency across space and time. Quantification of regional differences in boreal GSFDs provide insights regarding variations in ecological processes, including the manifestation of gap phase dynamics, as well as informing forest management practices that seek to emulate natural disturbance patterns.

## Data Availability

Representative ALS transects are freely available via Wulder *et al*.^56^. Computer code for computation of gap size frequency distributions are freely available and provided in Hanel *et al*.^58^.
